# Altered cardiac‐coronary coupling relates to abnormal fractional flow reserve without flow limitation after percutaneous coronary interventions

**DOI:** 10.14814/phy2.70440

**Published:** 2025-06-30

**Authors:** Ahmet Tas, Alp Ozcan, Yaren Alan, Sabahattin Umman, Kim H. Parker, Tim P. van de Hoef, Murat Sezer, Jan J. Piek

**Affiliations:** ^1^ Department of Cardiology Amsterdam UMC, Heart Centre, Amsterdam Cardiovascular Sciences Amsterdam the Netherlands; ^2^ Emergency Department Gomec State Hospital Balikesir Turkey; ^3^ Faculty of Medicine Istanbul University Istanbul Turkey; ^4^ Department of Cardiology Istanbul University Istanbul Turkey; ^5^ Department of Bioengineering Imperial College London UK; ^6^ Department of Cardiology University Medical Center Utrecht Utrecht the Netherlands; ^7^ Department of Cardiology Acibadem International Hospital Istanbul Turkey

**Keywords:** coronary artery disease, fractional flow reserve, hyperemic stenosis resistance, PCI, wave intensity analysis

## Abstract

A significant proportion of stenoses have an abnormal fractional flow reserve (FFR) after angiographically successful percutaneous coronary intervention (PCI), which is traditionally attributed to differences in coronary flow velocity reserve (CFVR) or hyperemic microvascular resistance (hMR). This study investigated the mechanisms underlying residual low FFR despite good angiographic results and normalized hyperemic stenosis resistance (hSR) using wave‐intensity analysis (WIA), which evaluates phasic characteristics of cardiac‐coronary coupling. Sixty‐three vessels from patients who underwent PCI for stable intermediate stenoses were included. Peri‐PCI characteristics of conventional and WIA parameters were assessed. Ten (16%) vessels exhibited residual low FFR (≤0.8) despite normalized hSR (<0.8), without significant differences in hyperemic flow (velocity) (hAPV), CFVR, or hMR compared with concordant normal FFR‐hSR group (*p* > 0.05). WIA revealed a significantly slower (peak‐time of backward expansion wave, tBEW_peak_ = 20% ± 6% vs. 29% ± 17% of expansion period, *p* = 0.005) and weaker (BEW_peak_ = 10.1 ± 8.9 vs. 15.8 ± 11.4 10.kW.m‐2.s‐2, *p* = 0.045) impact of microvascular suction responsible for diastolic coronary filling during hyperemia in the low FFR group, despite comparable pre‐PCI characteristics. Residual low FFR after PCI may reflect altered cardiac‐coronary coupling without limitation of flow or vasodilator capacity when the blunted impact of accelerating wave energy flux with delayed coronary filling fails to sustain distal pressure. The influence of cardiac‐coronary coupling on post‐PCI FFR warrants further investigation.

## 1 BACKGROUND

Following percutaneous coronary intervention (PCI), the primary objective is to restore normal coronary blood flow and alleviate anginal symptoms. However, a significant proportion of patients (~20%–40%) exhib persistent abnormalities in post‐PCI physiological parameters, such as fractional flow reserve (FFR), despite achieving angiographically satisfactory results, that may translate into a higher residual risk for clinical events (Masdjedi et al., 2022; Shin et al., 2020). The discrepancy between anatomical and functional assessment raises concerns about the completeness of revascularization and the potential for persistent myocardial ischemia even in the absence of visually apparent residual stenosis. Several potential mechanisms have been proposed to explain this mismatch between angiographic results and hemodynamic indices, including the presence of residual focal or diffuse stenosis (Bech et al., 1999), distal embolization of plaque debris (Ng et al., 2012), microvascular dysfunction (Van De Hoef et al., 2013), and altered vasomotor tone (Eftekhari et al., 2022; Fearon et al., 2013). However, the precise interplay of these factors and their contributions to post‐PCI physiological abnormalities, that is, residual low FFR despite angiographically good outcome, remains incompletely understood. Due to the inherently unique pressure–flow relationships in coronary arteries unlike other arterial beds, where the driving force (pressure, P) also limits the acceleration of the flow (velocity) during contraction resulting in a diastole‐dominant flow pattern, a true understanding of coronary hemodynamics requires simultaneous assessment of pressure (P) and flow velocity (U). This knowledge gap underscores the need for advanced diagnostic tools that can provide a more comprehensive assessment of coronary physiology. Large clinical studies have demonstrated that abnormalities of pressure‐indices are not necessarily clinically relevant if there is no significant limitation of the flow (van de Hoef et al., 2022, 2023). Yet, CFVR or flow/velocity‐only indices alone cannot be used for evaluation of an epicardial lesion, as the indices are highly sensitive to alterations of microvascular functionality, resistance and resting flow, beyond epicardial disease.

In this context, wave intensity analysis (WIA) emerges as a powerful tool because it simultaneously captures pressure changes generated by ventricular contraction/relaxation and the corresponding acceleration/deceleration in distal coronary flow (velocity). WIA characterizes the magnitude and direction of phasic wave energy transfer in the coronary circulation, allowing investigation of cardiac–coronary coupling. This method can potentially dissect the contributions of various physiological processes while distinguishing wave origins (proximal vs. distal) with their magnitudes and timings (Parker, 2009;  ). Previous research utilizing WIA in the setting of stable CAD and/or PCI has demonstrated that PCI augments the accelerating wave energy flux in general, but some cases do not exhibit an adequate response (Narayan et al., 2015). Unlike these paired analyses, studies in stable CAD demonstrate that hemodynamic parameters such as wave amplitudes show only poor or nonsignificant correlations with FFR, CFVR, or diameter stenosis percentage (DeMarchi et al., 2019). Recently, we analyzed 423 intermediate stenoses from 358 stable CAD patients and found that the temporal characteristics of net WI exhibited distinct differentiation among FFR/CFVR subgroups (Tas, Alan, et al., 2025). These findings suggest that WIA may meaningfully capture hemodynamic signatures of cardiac–coronary interactionthat are concealed by conventional indices like FFR or CFVR.

Therefore, this study aims to investigate the underlying pathophysiological and hemodynamic interplays in the setting of residually low FFR despite good angiographic results following PCI and normalized stenosis resistance, from the perspective of WIA and cardiac‐coronary‐coupling.

## 2 METHODS

This exploratory hemodynamic study retrospectively included available cases from pooled data from the DEFINE‐FLOW study (Dryad, n.d.; Stegehuis et al., 2020) and Amsterdam UMC, from patients who underwent coronary angioplasty for intermediate, stable stenoses. The analysis included temporally paired hemodynamic measurements from 63 vessels from 61 patients before and after PCI. The original study and the present analysis were conducted in accordance with the Declaration of Helsinki. The Institutional Ethical Review Board of Amsterdam UMC approved the original study, and all patients gave written informed consent.

### 2.1 Hemodynamic study

The detailed catheterization laboratory protocol can be found in detail elsewhere (Stegehuis et al., 2020). The “< >” denotes time‐averages of parameters to ensure the discrimination from continuous variables. Briefly, aortic pressure (Pa) was measured via a guiding catheter placed at the coronary ostium. Distal coronary pressure (Pd) and blood (flow) velocity were measured using a 0.014‐inch guidewire equipped with both a Doppler velocity probe and a pressure sensor (ComboWire, Volcano, San Diego, CA). The wire was positioned in the target vessel with both sensors located distally to the coronary stenosis. Pressure signals for DEFINE‐FLOW study were recorded at 200 Hz and velocity was recorded at 100 Hz. For other cases, all signals were recorded at 120 Hz. Measurements were obtained at rest and during hyperemia induced by adenosine (100 μg for left or right coronary arteries; 60 μg if atrioventricular block occurred), or intravenously as a continuous infusion at 140 μg/kg per minute, both of which can be interchangeably used in clinical practice, yielding identical results (Schlundt et al., 2015). Wave‐intensity analysis was made adjusting for the corresponding sampling‐frequencies (Parker, 2009).

### 2.2 Post‐processing for signal analysis

Doppler flow velocity signals were filtered with a second‐order Savitzky–Golay filter, with a window length of 11 samples. Resting and hyperemic time windows were automatically selected in MATLAB following maximum mean changes and approved via visual inspection for the quality of envelopes and correctness of the beginning of the hyperemic stimulus. Then, pressure and flow velocity waveforms in the selected time windows were ensemble‐averaged into a single representative cardiac cycle. This ensemble average beat enabled the analysis of temporal variations in the WI parameters during the beat; information that would otherwise be lost in the variance in signal‐averaged measurements.

### 2.3 Definitions of hemodynamic indices

Basal microvascular resistance (bMR) and hyperemic microvascular resistance (hMR) were calculated with basal (b) and hyperemic (h) mean flow velocity (APV) and pressure (P) in accordance with their definitions (bMR $=\frac{<\mathrm{bPd}>}{<\text{bAPV}>}$ and hMR = $\frac{<\mathrm{hPd}>}{<\text{hAPV}>}$). The fractional flow reserve (FFR) is calculated as the ratio of the mean distal pressure (Pd) to the mean pressure proximal to the lesion (Pa) (FFR = $\frac{<\mathrm{Pd}>}{<\mathrm{Pa}>}$) during hyperemia. The coronary flow velocity reserve (CFVR) is calculated by $\frac{<\text{hAPV}>}{<\text{bAPV}>}$. Hyperemic stenosis resistance (hSR) was defined as $\frac{<\left(\mathrm{Pa}-\mathrm{Pd}\right)>}{<\text{hAPV}>}$. The vessels were stratified as per the hSR and FFR with the cutoff value of 0.80 in line with the literature (Meuwissen et al., 2002; van de Hoef et al., 2023). Additionally, we computed microvascular resistance reserve [MRR = (CFVR/FFR) × (<Pa rest>/<Pa hyperemia>)] as a measure of microvascular vasodilatory capacity adjusted for epicardial disease severity (Boerhout et al., 2023; De Bruyne et al., 2021; de Vos et al., 2023). We also calculated the resistive reserve ratio (RRR = bMR/hMR), a marker of autoregulatory capacity with robust prognostic value (Toya et al., 2021).

### 2.4 Coronary wave intensity analysis

Invasively obtained coronary flow velocity and pressure data recorded at rest and during hyperemia were used to visualize and quantify the flow, pressure, and resistance‐based indices of epicardial and microvascular disease severity as well as the coronary WI.

**FIGURE 1:**
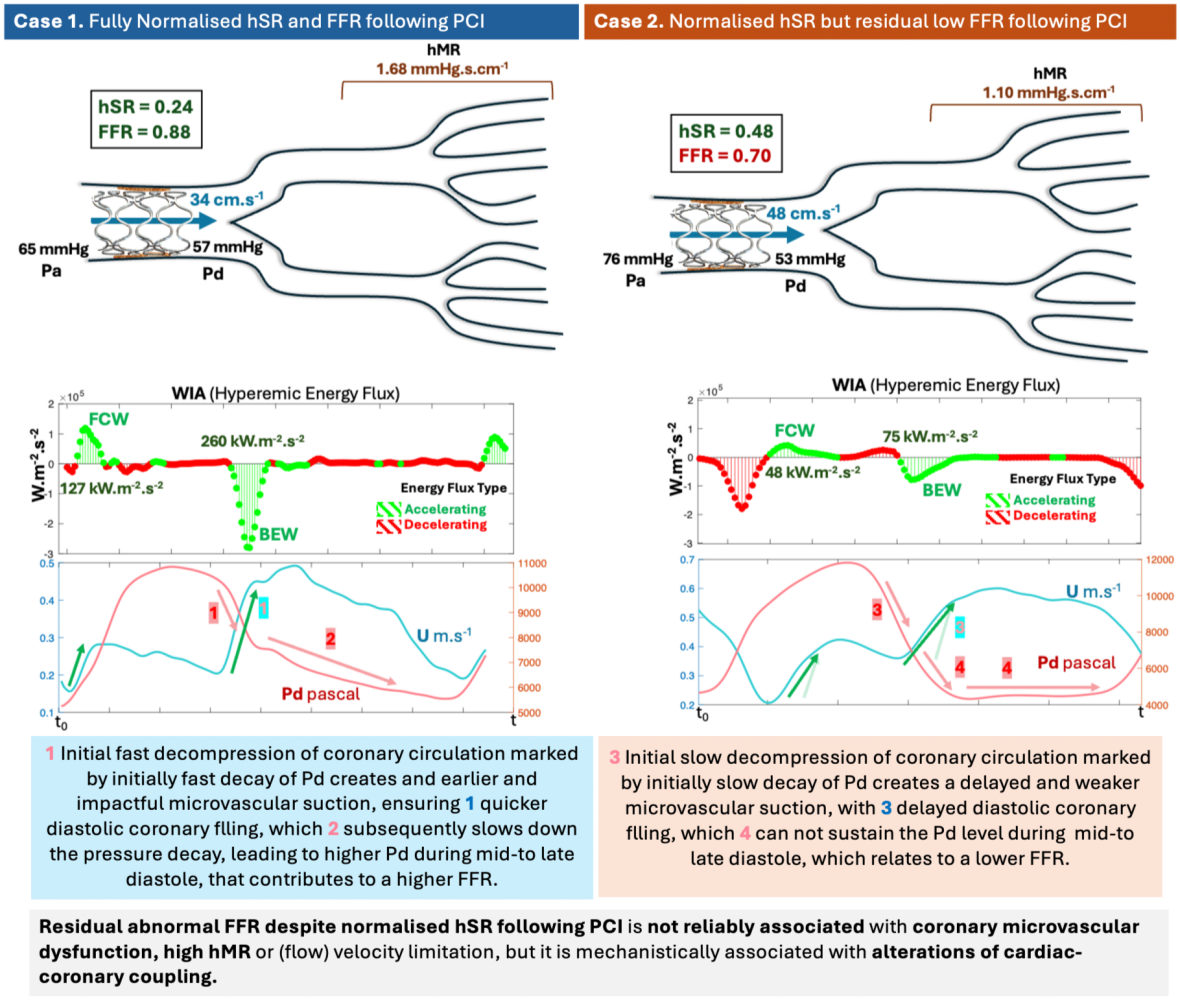
Visual Abstract Residual low fractional flow reserve (FFR) despite normal hyperemic stenosis resistance (hSR) following percutaneous coronary intervention (PCI) does not associate with impaired hyperemic flow (hAPV, hyperemic average peak velocity) or flow augmentation capacity (CFVR, coronary flow velocity reserve). As can be appreciated from the two example cases, the case on the right had similar distal coronary pressure (Pd) and numerically higher hAPV as well as lower hyperemic microvascular resistance (hMR) despite having a FFR of 0.70. This case has relatively lower peak backward expansion wave (BEW) and forward compression wave (FCW), which do not coincide with impaired perfusion parameters. Initial fast decompression of coronary circulation marked by initially fast decay of Pd creates an earlier and impactful microvascular suction, ensuring quicker diastolic filling, which subsequently slows down the pressure decay, leading to higher Pd during mid‐to late diastole, that relates to a higher FFR. Initial slow decompression of coronary circulation marked by initially slow decay of Pd creates a later and weaker microvascular suction, with delayed diastolic filling, which cannot sustain the Pd level during mid‐to late diastole, causing sooner an early nadir of Pd, that relates to a lower FFR.

WIA used to quantitatively evaluate the coronary arterial energy transfer characteristics was performed with an updated and fully automatized version of Kim H. Parker's dedicated software (Imperial College, UK) (Parker, 2009) used in our previous studies (Hasdemir et al., 2023; Tas, Alan, et al., 2025; Tas, Alp Findik, et al., 2025). Ensemble averaged arterial blood pressure and flow velocity signals as previously described (WI = (dP/dt)*(dU/dt), W.m‐2.s‐2) (Figure 1). The ensemble average was taken over the widest temporal frame with adequate signal quality, including no fewer than 8 consecutive beats in the present study. The variables of interest were the peak amplitudes of forward compression wave (FCW), backward compression wave (BCW), forward expansion wave (FEW), and backward expansion wave (BEW) (Broyd et al., 2017).

Because the BEW dominates the acceleration in the coronary arteries and is responsible for the diastolic coronary filling, we also measure the time of the BEW peak (Tas, Alan, et al., 2025). We defined the expansion phase of the beat as dP/dt <0, which extends from the systolic peak blood pressure to the end of diastole, covering the reduced ejection phase of late systole and the entire diastolic period. To quantify how quickly the expansion of the myocardium can exert its maximum impact on the coronary circulation, we measured the time between the start of the expansion phase (the time of peak systolic pressure) to the time of peak BEW. In order to compare patients with different heart rates, we expressed this time as a fraction of the total time of the expansion phase; and the relative time ,tBEW_peak_ , was expressed as a percentage (Tas, Alan, et al., 2025). All indices were calculated both at rest and during hyperemia.

### 2.5 Statistical analysis

Continuous variables are expressed as mean ± standard deviation. Normality of variables was assessed using the Shapiro–Wilk test. Means were compared using independent samples *t*‐test (Student's) or Mann–Whitney U test for independent variables or using paired samples *t*‐test (Student's) or Wilcoxon signed‐rank test for paired measurements. The categorical variables were compared using chi‐square or Fisher's exact tests, according to normality. Correlations were evaluated using Pearson's or Spearman's correlation coefficients when applicable. A *p* value <0.05 was considered statistically significant. All data were blindly analyzed offline using R‐based JAMOVI statistical software (The Jamovi Project, Sydney, Australia).

## 3 RESULTS

### 3.1 Demographics

The analysis included temporally paired hemodynamic measurements from 63 vessels in 61 patients before and after PCI. Table 1 presents the demographic characteristics for concordant (FFR > 0.80 and normal hSR post‐PCI) and discordant (residual low FFR ≤ 0.8 despite normal hSR post‐PCI) groups, and the entire group side‐by‐side. There were no significant differences between the concordant and discordant groups in terms of age, sex, comorbidities, or medications. Furthermore, patients with or without hypertension and/or diabetes had comparable pre‐ and post‐PCI WIA amplitudes both at rest and during hyperemia in all four calculated peaks (Tables S1 and S2). There was no significant difference between male and female sexes (Table S3).

**TABLE 1:** Patient‐level characteristics of residual low FFR (post PCI) cases.

	FFR > 0.8 (post‐PCI)	FFR ≤ 0.8 (post‐PCI)	Entire group	*p* Value
	*n* = 53 vessels	*n* = 10 vessels	*n* = 63 vessels	
	*n* = 51 patients	*n* = 10 patients	*n* = 61 patients	
Male sex	43.0 (81.1%)	8.0 (80.0%)	51.0 (81.0%)	0.933
Age, years	64.2 (9.6)	59.5 (6.9)	63.5 (9.3)	0.145
BMI, kg/m^2^	25.6 (3.7)	26.7 (4.8)	25.8 (3.8)	0.442
Cardiovascular risk factors				
Hypertension	26.0 (50.0%)	4.0 (40.0%)	30.0 (48.4%)	0.562
Diabetes mellitus	19.0 (35.8%)	2.0 (20.0%)	21.0 (33.3%)	0.329
Smoking	35.0 (67.3%)	4.0 (44.4%)	39.0 (63.9%)	0.187
Dyslipidemia	48.0 (90.6%)	7.0 (70.0%)	55.0 (87.3%)	0.199
Medications				
Beta‐blockers	38.0 (71.7%	7.0 (70.0%)	45.0 (71.4%)	0.913
Calcium antagonists	24.0 (45.3%)	4.0 (40.0%)	28.0 (44.4%)	0.758
Insulin	3.0 (5.7%	1.0 (10.0%)	4.0 (6.3%)	0.606
Oral antidiabetics	12.0 (22.6%)	1.0 (10.0%)	13.0 (20.6%)	0.365
Nitrates	23.0 (43.4%)	2.0 (20.0%)	25.0 (39.7%)	0.165
RAAS inhibitors	18.0 (34.6%)	1.0 (10.0%	19.0 (30.6%	0.122
Acetylsalicylic acid	48.0 (92.3%)	9.0 (90.0%)	57.0 (91.9%)	0.806

*Note*: Missing data: Hypertension *n* = 1 in concordant group, Smoking: *n* = 1 in concordant and *n* = 1 in discordant group; ACEi *n* = 1 in concordant group, ASA *n* = 1 in concordant group. Study groups have two patients in common, each of whom had two revascularized vessels.

### 3.2 Baseline hemodynamic characteristics and peri‐PCI changes

Table 2 demonstrates hemodynamic parameters before and after PCI. PCI induced significant improvements in FFR (0.64 ± 0.15 vs. 0.87 ± 0.07, *p* < 0.001), hSR (1.71 ± 1.77 vs. 0.26 ± 0.18 mmHg.cm‐1.s, *p* < 0.001), and CFVR (1.67 ± 0.42 vs. 2.47 ± 0.64, *p* < 0.001), with significant augmentation of hAPV (29.3 ± 18.2 vs. 48.7 ± 17.4 cm.s‐1, *p* < 0.001). bAPV increased slightly (18.39 ± 9.96 vs. 20.86 ± 7.85 cm.s‐1, *p* = 0.016) without a statistically significant mean change in bMR (4.89 ± 2.07 vs. 4.91 ± 1.96 mmHg.cm‐1.s, *p* = 0.591).

**TABLE 2:** Peri‐PCI hemodynamic characteristics.

		*N*	Mean	Median	SD	*p* Value
FFR_pre_	Unitless	63	0.64	0.67	0.15	<0.001***
FFR_post_		63	0.87	0.89	0.07	
CFVR_pre_		63	1.67	1.54	0.42	<0.001***
CFVR_post_		63	2.47	2.48	0.64	
hSR_pre_	mmHg.cm^−1^.s	63	1.71	1.06	1.77	<0.001***
hSR_post_		63	0.26	0.22	0.18	
bMR_pre_		63	4.89	4.35	2.07	0.591
bMR_post_		63	4.91	4.68	1.96	
hMR_pre_		63	2.43	2.11	1.05	<0.001***
hMR_post_		63	1.78	1.63	0.72	
bAPV_pre_	cm.s^−1^	63	18.39	16.54	9.96	0.016*
bAPV_post_		63	20.86	18.72	7.85	
hAPV_pre_		63	29.28	26.13	18.18	<0.001***
hAPV_post_		63	48.66	45.99	17.44	

*Note*: “Pre” subscription indicates pre‐PCI, whereas “post” indicates post‐PCI measurements.

Abbreviations: bAPV, basal average peak velocity; bMR, basal microvascular resistance; CFVR, coronary flow velocity reserve; FFR, fractional flow reserve; hAPV, hyperemic average peak velocity; hMR, hyperemic microvascular resistance; hSR, hyperemic stenosis resistance.

*p < 0.05, **p < 0.01, ***p < 0.001.

### 3.3 Hemodynamic characteristics of residual low FFR despite normalized hSR

There were 10 cases (~16%) with residual low FFR (≤0.8) post‐PCI despite a normalized hSR. These lesions had angiographically less severe stenoses indicated by mean lower DS% before PCI (74% ± 14% vs. 64% ± 14%, *p* = 0.034), but hemodynamic characteristics were similar. The magnitudes of PCI‐induced improvements were similar in terms of hAPV (19.3 ± 23.7 vs. 20.0 ± 16.6 cm.s‐1, *p* = 0.921), CFVR (0.83 ± 0.67 vs. 0.71 ± 0.72, *p* = 0.529), and hSR (1.52 ± 1.88 vs. 1.04 ± 1.25 mmHg.cm‐1.s, *p* = 0.458) for the two groups, despite less pronounced FFR augmentation (0.13 ± 0.12 vs. 0.26 ± 0.17, *p* = 0.042) in the residual low FFR group (The latter values in parentheses correspond to the residual low FFR group).

Compared to cases with concordantly normalized post‐PCI hSR and FFR, the residual low FFR group had relatively higher mean hSR—although all values were within the normal range—(0.21 ± 0.13 vs. 0.52 ± 0.17 mmHg.cm‐1.s, *p* < 0.001), but no significant limitation of flow or vasodilatory capacity marked by indifferent means of bAPV (21.0 ± 8.3 vs. 20.2 ± 5.3 cm.s‐1, *p* = 0.779), hAPV (48.7 ± 18.2 vs. 48.4 ± 13.5 cm.s‐1, *p* = 0.964), and CFVR (2.48 ± 0.66 vs. 2.45 ± 0.52, *p* = 0.888). The two groups had similar mean microvascular resistance during rest (bMR; *p* = 0.281) or hyperemia (hMR; *p* = 0.152) (Table 3). In addition to the comparable CFVR, the residual low FFR group did not have worse microvascular vasodilator capacity or microvascular functionality, as shown by comparable MRR and RRR on following PCI (Table 3).

**TABLE 3:** Lesion‐level characteristics of residual low FFR (post PCI) cases.

	FFR^a^	N	Mean/n	SD/%	*p* Value
RCA‐Lesion	>0.8	53	10	18.9%	0.134
	≤0.8	10	0	0%	
DS%	>0.8	53	74.3	13.8	0.034*
	≤0.8	10	63.8	14.4	
FFR_pre_	>0.8	53	0.64	0.15	0.275
	≤0.8	10	0.61	0.14	
CFVR_pre_	>0.8	53	1.65	0.39	1.000
	≤0.8	10	1.73	0.57	
hSR_pre_	>0.8	53	1.73	1.86	0.714
	≤0.8	10	1.55	1.29	
bMR_pre_	>0.8	53	4.87	2.01	0.977
	≤0.8	10	4.99	2.46	
hMR_pre_	>0.8	53	2.48	1.10	0.554
	≤0.8	10	2.13	0.68	
bAPV_pre_	>0.8	53	18.64	10.45	0.800
	≤0.8	10	17.09	7.06	
hAPV_pre_	>0.8	53	29.44	18.94	0.962
	≤0.8	10	28.39	14.29	
MRR_pre_	>0.8	53	2.75	0.68	0.247
	≤0.8	10	2.97	0.66	
RRR_pre_	>0.8	53	2.05	0.55	0.305
	≤0.8	10	2.35	0.77	
FFR_post_	>0.8	53	0.90	0.04	<0.001***
	≤0.8	10	0.73	0.06	
CFVR_post_	>0.8	53	2.48	0.66	0.888
	≤0.8	10	2.45	0.52	
hSR_post_	>0.8	53	0.21	0.13	<0.001***
	≤0.8	10	0.52	0.17	
bMR_post_	>0.8	53	5.02	2.04	0.281
	≤0.8	10	4.29	1.35	
hMR_post_	>0.8	53	1.84	0.75	0.152
	≤0.8	10	1.48	0.53	
bAPV_post_	>0.8	53	20.98	8.27	0.779
	≤0.8	10	20.22	5.30	
hAPV_post_	>0.8	53	48.71	18.20	0.964
	≤0.8	10	48.43	13.45	
MRR_post_	>0.8	53	3.08	0.79	0.102
	≤0.8	10	3.59	0.83	
RRR_post_	>0.8	53	2.81	0.73	0.485
	≤0.8	10	2.98	0.65	

*Note*: Results are presented as mean ± SD or *n* (%). “Pre” subscription indicates pre‐PCI, whereas “post” indicates post‐PCI measurements.

Abbreviations: bAPV, basal average peak velocity; bMR, basal microvascular resistance; CFVR, coronary flow velocity reserve; DS%, stenosis diameter; FFR, fractional flow reserve; hAPV, hyperemic average peak velocity; hMR, hyperemic microvascular resistance; hSR, hyperemic stenosis resistance; RCA, right coronary artery.

^a^ Post PCI FFR.

*p < 0.05; **p < 0.01; ***p < 0.001.

**FIGURE 2:**
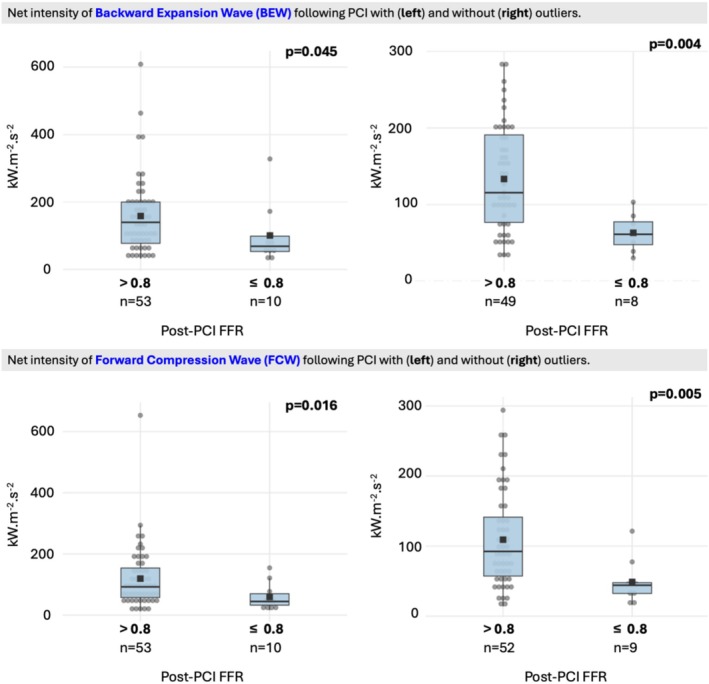
Residual low FFR after PCI is associated with lower hyperemic accelerating wave energy flux during systole and diastole despite comparable mean velocity characteristics highlighting the distinct phasic differences in cardiac‐coronary coupling (data is expressed as mean ± SD).

In contrast to the similar impact on flow (velocity) during rest or hyperemia, vessels with residual low FFR despite normalized hSR had significantly lower accelerating wave energy flux during compression (FCW = 11.9 ± 10.2 vs. 5.9 ± 4.5 10.kW.m‐2.s‐2, *p* = 0.016) and expansion (BEW = 15.8 ± 11.4 vs. 10.1 ± 8.9 10.kW.m‐2.s‐2, *p* = 0.045) periods during hyperemia without pronounced differences during rest or in decelerating wave peaks (i.e., BCW and FEW) (Figure 2). Accordingly, the accelerating wave energy proportion was lower in the discordant group than in the concordant group (62% ± 16% vs. 50% ± 16%, *p* = 0.035) and the peak accelerating impact of microvascular suction was delayed during hyperemia (tBEW_peak_: 20% ± 6% vs. 29% ± 17%, *p* = 0.005) in the residual low FFR group whereas both pre‐ and post‐PCI resting tBEW_peak_ and hyperemic tBEW_peak_ were similar (Table 4). Importantly, the discordant and concordant groups had no statistically significant differences in mean amplitudes of net WI before PCI during rest or hyperemia (Table 4), indicating the observed differences were not simply due to pre‐existent abnormalities before the PCI.

**TABLE 4:** Differences of net wave intensity analysis pre and post‐PCI.

	Variable	FFR^a^	Mean	SD	*p* Value	Variable	FFR*	Mean	SD	*p* Value
Rest	FCW_pre_	>0.8	6.5	8.5	0.652	FCW_post_	>0.8	7.0	6.0	0.700
		≤0.8	3.7	2.3			≤0.8	5.2	3.1	
	BCW_pre_	>0.8	5.2	5.4	0.814	BCW_post_	>0.8	5.8	5.0	0.814
		≤0.8	5.4	4.4			≤0.8	4.8	2.4	
	FEW_pre_	>0.8	3.2	3.7	0.645	FEW_post_	>0.8	4.6	4.9	0.785
		≤0.8	3.0	3.6			≤0.8	3.3	2.4	
	BEW_pre_	>0.8	10.9	13.0	0.948	BEW_post_	>0.8	13.2	14.7	0.821
		≤0.8	8.1	5.0			≤0.8	9.8	7.0	
	tBEW_pre_	>0.8	0.23	0.10	0.337	tBEW_post_	>0.8	0.17	0.05	0.792
		≤0.8	0.20	0.13			≤0.8	0.18	0.08	
Hyperemia	FCW_pre_	>0.8	7.1	9.7	0.129	FCW_post_	>0.8	11.9	10.2	0.016*
		≤0.8	2.3	1.1			≤0.8	5.9	4.5	
	BCW_pre_	>0.8	5.7	5.4	0.700	BCW_post_	>0.8	11.6	10.4	0.888
		≤0.8	5.5	4.4			≤0.8	9.9	5.9	
	FEW_pre_	>0.8	3.2	3.7	0.566	FEW_post_	>0.8	6.2	6.0	0.446
		≤0.8	2.6	2.4			≤0.8	4.6	3.9	
	BEW_pre_	>0.8	8.9	11.4	0.219	BEW_post_	>0.8	15.8	11.4	0.045*
		≤0.8	4.4	3.1			≤0.8	10.1	8.9	
	tBEW_pre_	>0.8	0.28	0.10	0.579	tBEW_post_	>0.8	0.20	0.06	0.005**
		≤0.8	0.34	0.13			≤0.8	0.29	0.17	

*Note*: Results are presented as mean ± SD. “Pre” subscription indicates pre‐PCI, whereas “post” indicates post‐PCI measurements. WI peaks have the unit of 10.kW.m^−2^.s^−2^. *n* = 10 for FFR ≤ 0.8 and *n* = 53 for FFR > 0.8.

Abbreviations: BCW, backward compression wave; BEW, backward expansion wave; FCW, forward compression wave; FEW, forward expansion wave; tBEW, relative time of peak backward expansion wave.

^a^ Post PCI FFR.

*p < 0.05; **p < 0.01; ***p < 0.001.

### 3.4 Correlation between WIA amplitudes and epicardial disease severity pre‐ and post‐PCI

Mild‐to‐moderate correlations were present between microvascular resistance (bMR and hMR), distal blood velocity (bAPV and hAPV), stenosis resistance (hSR), and WI amplitudes during rest and hyperemia both before and after PCI. Attenuation of epicardial stenosis after PCI altered the pre‐PCI correlations between CFVR and FFR and WI amplitudes. In the post‐PCI state, the hyperemic amplitudes of FCW and BEW showed moderate correlations with hMR, hSR, and hAPV. Compared to these, less pronounced correlations were present between FFR and post‐PCI hyperemic FCW but not BEW (Table S4).

### 3.5 Interrelation between use of absolute difference (∆P) or ratio between proximal and distal coronary pressure

**FIGURE 3:**
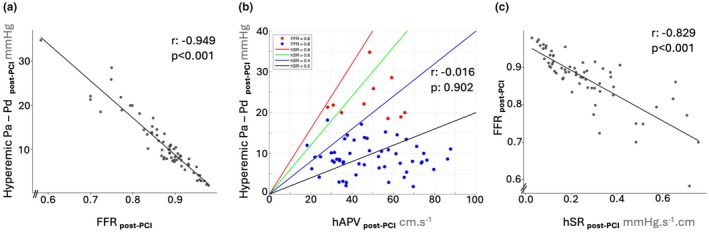
FFR—hSR discordance is not primarily associated with formulation difference and use of ∆P (Pa – Pd) versus Pd/Pa, or an increased flow (velocity). There were discordant and concordant cases with similar ∆P and similar hAPV within the same hSR range.

The absolute difference (∆P = Pa – Pd, utilized in the hSR calculation) and the ratio of Pd/Pa (employed in the FFR calculation) exhibited a robust correlation with each other (*r* = −0.949, *p* < 0.001) (Figure 3a). This finding challenges the notion that this formulation difference plays a primary role in the discordance between FFR and hSR. In addition, although the mean hSR was higher in the discordant group, all cases had hSR below 0.80 (red line—Figure 3b) and there were concordant and discordant cases with hSR values between hSR ranges of 0.20–0.40, 0.40–0.60, and 0.60–0.80 (Figure 3b,c). Moreover, higher mean hyperemic flow was not unequivocally associated with increased hyperemic translesional pressure difference (∆P) (*r* = −0.016, *p* = 0.902) (Figure 3b), and there were discordant and concordant cases with similar ∆P and similar hAPV (Figure 3b), which was in line with the indifferent mean hAPV of discordant and concordant lesions.

### 3.6 Impact of heart rate

The heart rate‐adjusted WI profiles compared between the discordant and concordant groups yielded parallel results with the main analysis (Tables S5 and S6).

## 4 DISCUSSION

In this study, we examined the residual low FFR despite angiographically good results and normalized hSR following elective PCI for stable intermediate coronary stenoses. For this purpose, we evaluated the phasic differences of cardiac‐coronary coupling via coronary WIA in addition to the traditional hemodynamic parameters. The principal findings of this study are as follows:

(1) Following PCI, the cases with a residual low FFR despite angiographically good outcome tend to have higher epicardial stenosis resistance (hSR) without appreciable differences in the resting (bMR) or hyperemic (hMR) microvascular resistance. Although PCI attenuated hSR to the normal range with comparable improvement in all cases, relatively higher hSR with similar hMR values resulted in lower FFR, consistent with the alternative, resistance‐based formulation of FFR = $\frac{\mathrm{hMR}\;}{\mathrm{hMR}+\mathrm{hSR}}$ (Eftekhari et al., 2022). These cases had lower amplitudes of accelerating wave energy flux during hyperemia (FCW and BEW) with a relatively delayed peak impact of microvascular suction effect (tBEW) which is mainly responsible for the diastolic coronary filling. Nonetheless, the normal hSR values accompanied by intact vasodilatory response (MRR/CFVR) and autoregulatory capacity (RRR), which are statistically indifferent compared to the FFR > 0.80 group, ensured that there was no limitation of flow (velocity) during rest (bAPV) or hyperemia (hAPV).

(2) These findings challenge the conventional explanations for the residual low FFR phenomenon, as the discordance was not due to distal microembolization (similar hMR and bMR post‐PCI), worse microvascular function (similar CFVR, MRR, and RRR) or altered vasomotor tone (similar hMR, bMR, and RRR), and not explainable by diffuse coronary disease (which typically yields preserved FFR with impaired CFVR, the opposite of our scenario). Yet, WIA revealed significantly lower accelerating wave energy flux with a delayed impact, suggesting that differences in cardiac‐coronary coupling could explain the low FFR group. Specifically, while a more pronounced and early impact of microvascular suction ensures an early and pronounced diastolic coronary filling—hence the recruited volume load may prevent an abrupt and early pressure loss in the distal coronary segments—it ensures a more gradual pressure decay during the mid‐to‐late diastole. Thus, Pd/Pa averaged over the cardiac cycle (and FFR) tends to remain higher. In contrast, a delayed and less impactful diastolic coronary filling cannot sustain the Pd during mid‐to‐late diastole and does not prevent the abrupt diastolic pressure drop; thus, the earlier nadir of distal pressure during diastole reflects lower values of the FFR. Although an initially fast pressure decay during the expansion period relates to an earlier and stronger BEW, this does not necessarily mean lower distal pressure throughout diastole, since the subsequent pronounced diastolic coronary filling smooths the pressure decay. Conversely, an initially more gradual pressure decay with delayed and less impactful BEW cannot drive the same impact (Visual Abstract). Although the long‐term significance of the differences in the cardiac‐coronary coupling and higher‐normal hSR is unknown, they do not appear to translate into limitation of flow (velocity) or impairment of vasodilatory response immediately after PCI reassuring the success of the intervention.

### 4.1 Characteristics and implications of an abnormal FFR following PCI

An abnormal FFR despite an angiographically normal coronary artery may be seen in the presence of residual focal or diffuse stenosis (Bech et al., 1999), distal embolization of plaque debris (Ng et al., 2012), microvascular dysfunction (Van De Hoef et al., 2013), and altered vasomotor tone (Eftekhari et al., 2022; Fearon et al., 2013). Diffuse coronary atherosclerosis may cause a gradual loss of pressure within an artery (i.e., FFR), without angiographically appreciable manifestations (De Bruyne et al., 2001). In cases of a hemodynamically significant focal stenosis superimposed on diffuse disease, limitation of flow by the proximally located obstruction may mask the severity of diffuse disease, yet, once the impact of focal lesion is resolved, the restored hyperemic flow unmasks the hemodynamic consequence of the diffuse disease, causing lower FFR values despite PCI (Hoshino et al., 2019). On the other hand, PCI‐induced distal microembolization causes plugging of the coronary microvascular bed with plaque debris, acutely increasing the microvascular resistance in the affected territories, yet compensatory vasodilation in neighboring vascular territories causing overall hyperemia and low FFR preventing a monotonic relationship, which may have contributed the indifferent mean hMR values in the study groups. The structural damage to the microcirculation is further complicated by the functional deterioration due to exhaustion of autoregulatory mechanisms because of chronic ischemia. In the post‐PCI setting, altered vasomotor tone is multifactorial and microvascular resistance may be abnormally low (e.g., due to post‐occlusive hyperemia, or inflammatory stimuli) or exaggeratedly high (e.g., due to microvascular plugging, or vasospasm from a burst of vasoactive substances). Both extremes of vasotonus abnormalities are associated with inadequate flow reserve in the PCI setting, because loss of resting vasotonus (bMR) is also associated with increased resting flow, blunting the CFVR, and an abnormally high microvascular resistance limits the hyperemic flow, once again blunting the CFVR (De Maria et al., 2019). Thus, coronary microvascular dysfunction (CMD), characterized by a lower CFVR, may emerge as a final pathway in post‐PCI pathophysiology and is associated with significantly worse prognosis (De Maria et al., 2019; Hasdemir et al., 2023).

In the present study, the residual low FFR group did not demonstrate any appreciable differences in bMR or hMR, indicating that the discordance did not simply stem from differences in altered vasotonus or distal thrombo‐embolization plugging the microcirculation. Likewise, comparably preserved vasodilatory capacity evidenced by statistically indifferent MRR, CFVR, and RRR rules out the primary contribution of coexisting or de‐novo (iatrogenic) CMD. Although the discordant cases were mostly those with >20 mmHg hyperemic translesional pressure loss, and the mean hSR was close to the higher end of the normal range, and the residual abnormal FFR group had mean hSR higher than those with normal FFR, the abnormal FFR cases were scattered uniformly in hSR ranges from 0.20 to 0.80, also with large variance of hAPV indicating that the observation was not solely due to higher hSR values or higher hAPV.

There were, however, remarkable differences in the characteristics of cardiac‐coronary coupling, revealed by the WIA. The residual low FFR was associated with slower exertion of peak accelerating impact of microvascular decompression (marked by higher tBEW_peak_ values) as well as relatively lower amplitudes of accelerating wave energy flux in coronary arteries. Unlike the conventional parameters (APV, CFVR, and hMR) which do not account for the instantaneous and directional dynamic coupling between contraction and relaxation of ventricular myocardium and distal coronary flow, WIA profiling the characteristics of cardiac‐coronary coupling substantially varied between study groups. In the reference (normal FFR and hSR) level of cardiac‐coronary coupling, initial fast decompression of the coronary circulation, marked by an initially rapid decay of Pd, creates an earlier and impactful microvascular suction. This appears to ensure quicker diastolic coronary filling; hence, the recruited blood volume may subsequently slow down the pressure decay, leading to sustained Pd during mid‐to‐late diastole and correlating with a higher FFR. Conversely, in cases with observed alterations of cardiac‐coronary coupling, initial slow decompression of the coronary circulation, marked by an initially slow decay of Pd, creates a later and weaker microvascular suction with delayed diastolic coronary filling. This cannot sustain the Pd level during mid‐to‐late diastole, causing an earlier nadir of Pd and correlating with a lower FFR. On the other hand, unlike the expansion phase, the limitation in accelerating energy flux during compression may still be more dependent on the residual higher‐normal hSR, as there is a > 20 mmHg loss compared to the aortic root. Although the amplitudes of WI peaks are pronouncedly affected by the microvascular tonus and are not specific for the epicardial disease severity (Tas, Alan, et al., 2025) (DeMarchi et al., 2019), they provide valuable insights into cardiac‐coronary coupling in different scenarios. The accelerating wave amplitudes (i.e., FCW and BEW) attenuate with experimental proximal occlusion (DeMarchi et al., 2019) and improve with the PCI (Narayan et al., 2015). Narayan et al. previously reported that lesions with the greatest flow limitation (as indicated by lower pre‐PCI FFR and CFVR) had the most significant increases in wave intensity, highlighting a degree of heterogeneity where dynamic coronary flow recovery becomes important. In the present study, we have seen that normalization of epicardial resistance by PCI is accompanied by the attenuation of pre‐existing correlations between FFR or CFR with the amplitudes of accelerating waves of WI, where the relationship between microvascular resistance and WI amplitudes became more prominent compared to pre‐PCI state. This may be attributed to the contribution of total vascular resistance by hSR versus hMR being altered with the PCI, with an increased proportion of hMR. Unlike the amplitudes of WI, tBEW_peak_ is a parameter that is remarkably less dependent on the microvascular resistance but may reflect the combined impact of an upstream stenosis on the translesional pressure drop and vasodilatory capacity on distal flow (velocity) acceleration (Tas, Alan, et al., 2025).

The similar magnitude and temporal characteristics of WI profiles in the pre‐PCI state suggest that alterations of cardiac‐coronary interactions may be induced by the PCI procedure itself, or the restoration of flow and flow (velocity) reserve unmasked the previously concealed differences. The numerically lower, though not statistically significant, pre‐PCI WI peaks may indicate that at least in some cases PCI may have acutely exacerbated pre‐existing variations in cardiac‐coronary coupling. Importantly, as per the study protocol, there were no patients with severe LV hypertrophy, septal wall thickness at echocardiography of >13 mm or a severe cardiomyopathy defined by LV ejection fraction <30%. Although the present study did not incorporate LV physiology into our analysis, the use of temporally paired comparison of WIA characteristics may enable a sound attribution of the changes to the PCI procedure.

Given that flow is not affected, flow augmentation capacity is preserved, and autoregulatory capacity is excellent (on the basis of CFVR, MRR, and RRR), there appears to be no evidence in favor of additional maneuvers following PCI for the target lesion, although the prognostic relevance of differences in cardiac‐coronary coupling should be elucidated in future studies. Without incorporation of flow (velocity) into evaluation, FFR does not seem to fully capture the dynamic interplay and impact of a coronary lesion, which has been increasingly demonstrated and recognized in prognostic studies. DEFINE‐FLOW and ILIAS registry recommended a conservative approach for patients with preserved CFVR despite abnormal FFR, while only the concordantly low CFVR and FFR group required revascularization, based on the similar outcomes compared to PCI (van de Hoef et al., 2022, 2023). Furthermore, ILIAS indicates a favorable prognosis for those with normal hSR (Boerhout et al., 2024). Although previous studies reported that residual abnormal FFR is linked to the risk for future repeat revascularization and a worse prognosis (Masdjedi et al., 2022; Shin et al., 2020), in these studies, the residual low FFR group associated with worse outcomes had the lowest CFVR, which was driven by increased resting flow despite similar hyperemic flow, which indicates that the main pathology was not the hyperemic flow limitation; hence, residual obstruction due to stenosis could be involved (Shin et al., 2020). Similarly, in a series of 466 patients with chronic coronary syndrome with single‐vessel disease who underwent pre‐ and post‐PCI FFR and CFVR measurements, abnormal CFVR or FFR following intervention was associated with worse outcomes. The lesions with (*n* = 48) and without (*n* = 418) target vessel failure had mean FFR of 0.85 versus 0.87, indicating only marginal FFR differences. On the other hand, these groups had a remarkable difference in mean CFVR (1.94 vs. 2.85) despite indifferent hyperemic microvascular resistance values (Ueno et al., 2024). It is obvious that residual abnormalities of coronary flow following PCI translate into clinical deterioration, despite only marginal differences in FFR which were both above the traditional cut‐off related to ischemia.

Normal‐range epicardial resistance, the absence of flow or flow reserve limitation, and intact vasodilatory response with preserved microvascular functionality cumulatively indicate a good outcome of PCI, discouraging further intervention, where it is not clear whether attempts to further attenuate (already normalized) hSR translate into improved cardiac‐coronary coupling, and yield additional improvement of the already equi‐reference flow, flow reserve, and autoregulatory capacity. Although wire‐based pressure indices provide convenient proxy tools for physiological assessment, the incorporation of flow (velocity) measurements (e.g., via hSR or combined FFR and CFR) is crucial in post‐PCI assessment, as the clinical outcome is determined by the effect of the lesion on blood flow to the myocardium. Prognostic relevance of the findings of the present study, including the differences of cardiac‐coronary coupling, warrants further research.

## 5 CONCLUSION

In stable coronary artery disease, residual FFR impairment post‐PCI, despite normalized hSR, may reflect alterations of cardiac‐coronary coupling characteristics, where blunted and delayed impact of accelerating wave energy flux with delayed coronary filling fails to sustain distal pressure. These cases do not necessarily exhibit microvascular dysfunction, variance in the microvascular resistance, limitation of hyperemic flow, or lower vasodilatory capacity. In the absence of flow‐limitation and impairment of vasodilatory response, a residual low FFR does not unequivocally indicate a poor outcome of PCI. This phenomenon underscores the multidimensional nature of coronary physiology and advocates for a holistic assessment framework that integrates flow (velocity) and pressure‐based assessment, such as WIA, and to optimize post‐revascularization evaluation of coronary physiology and to avoid unnecessary interventions.

### 5.1 Limitations

This is a hypothesis‐generating hemodynamic study with several limitations. First, the small sample size (*n* = 10 residual low FFR cases) limits statistical power and generalizability, and WIA‐derived parameters are not yet standardized in clinical practice, complicating immediate translation. The study did not incorporate stenoses geometry (e.g., diffuse or focal plaque distribution) and its potential influence. We also could not directly assess left ventricular function or peri‐procedural myocardial injury markers (such as ejection fraction or troponin levels); such data could help differentiate whether subtle LV dysfunction contributed to the observed changes in cardiac coronary coupling. Finally, long‐term outcomes were not assessed; thus, the prognostic significance of post‐PCI cardiac–coronary coupling differences remains unclear.

### 5.2 Future directions

Correlating WIA parameters with clinical endpoints (e.g., angina relief and target vessel failure) could refine their prognostic value. Technical advancements to simplify WIA integration into routine angiography may facilitate broader adoption. Furthermore, future studies could investigate treatments, including pharmacological agents and mechanical interventions, that could modulate cardiac‐coronary coupling to determine if WIA‐defined abnormalities can be alleviated.

## CONFLICT OF INTEREST STATEMENT

TvdH has received speaker fees and institutional research grants (not related to present work) from Abbott and Philips. JJP has received support as consultant for Philips/Volcano and has received institutional research grants from Philips (not related to present work). The other authors report no relationship with industry related to this work. The paper is not under consideration elsewhere and none of the paper's contents have been previously published. All authors have read and approved the manuscript.

## FUNDING INFORMATION

None.

## ETHICS STATEMENT

The original study and the present analysis were conducted in accordance with the Declaration of Helsinki. Institutional Ethical Review Board of Amsterdam UMC approved the original study, and all patients gave written informed consent.

## Supporting information


Tables S1–S6.

## Data Availability

All signal tracings as well as clinical information of DEFINE‐FLOW study are publicly available from https://datadryad.org/dataset/doi:10.5061/dryad.h18931zm6. The signal tracings of additional cases from the Amsterdam UMC may be requested from the lead author (AT), but immediate access is not warranted and may be subject to institutional approval. The custom software used in the present study is a modified version based on the MATLAB code written by Kim H Parker, presented in https://kparker.bg‐research.cc.ic.ac.uk/guide_to_wia/00_introduction.html in detail. The technical details and software sections have been modified in several positions. For technical questions, correspondence will be available from the lead author (AT).
