# Supplementary material for: Altered cardiac‐coronary coupling relates to abnormal fractional flow reserve without flow limitation after percutaneous coronary interventions
Source: Physiol Rep. 2025 Jun 30;13(13):e70440. doi: 10.14814/phy2.70440 (PMC12208797; doi:10.14814/phy2.70440)
Supplement: Supplementary file 1 — Tables S1–S6. [file PHY2-13-e70440-s001.docx]

**Supplementary Material**

**Supplementary Material: Impact of HT**

| **Supplementary Table 1.**  Hypertension (HT)$-$no HT Differences of Net Wave Intensity Analysis | | | | | | | | | | |
| --- | --- | --- | --- | --- | --- | --- | --- | --- | --- | --- |
|  | **Variable** | **HT** | **Mean** | **SD** | **P value** | **Variable** | **HT** | **Mean** | **SD** | **p value** |
| **rest** | **FCW_pre_** | **-** | 4.7 | 5.2 | 0.410 | **FCW_post_** | **-** | 7.0 | 5.1 | 0.257 |
|  |  | **+** | 6.8 | 9.2 |  |  | **+** | 6.5 | 6.3 |  |
|  | **BCW_pre_** | **-** | 4.9 | 4.3 | 0.972 | **BCW_post_** | **-** | 5.8 | 5.1 | 0.839 |
|  |  | **+** | 5.5 | 6.1 |  |  | **+** | 5.5 | 4.3 |  |
|  | **FEW_pre_** | **-** | 2.8 | 2.2 | 0.742 | **FEW_post_** | **-** | 5.4 | 4.9 | **0.014*** |
|  |  | **+** | 3.6 | 4.8 |  |  | **+** | 3.5 | 4.1 |  |
|  | **BEW_pre_** | **-** | 10.3 | 12.3 | 0.742 | **BEW_post_** | **-** | 12.4 | 10.7 | 0.390 |
|  |  | **+** | 10.0 | 11.9 |  |  | **+** | 13.3 | 16.8 |  |
| **hyperemia** | **FCW_pre_** | **-** | 4.9 | 6.9 | 0.309 | **FCW_post_** | **-** | 9.8 | 6.3 | 0.796 |
|  |  | **+** | 6.8 | 9.5 |  |  | **+** | 12.2 | 12.5 |  |
|  | **BCW_pre_** | **-** | 5.8 | 4.7 | 0.381 | **BCW_post_** | **-** | 12.9 | 10.7 | 0.184 |
|  |  | **+** | 5.6 | 5.9 |  |  | **+** | 9.7 | 8.7 |  |
|  | **FEW_pre_** | **-** | 3.0 | 2.7 | 0.711 | **FEW_post_** | **-** | 6.3 | 5.6 | 0.213 |
|  |  | **+** | 3.1 | 4.2 |  |  | **+** | 5.5 | 6.1 |  |
|  | **BEW_pre_** | **-** | 7.4 | 10.0 | 0.265 | **BEW_post_** | **-** | 15.4 | 10.7 | 0.619 |
|  |  | **+** | 8.9 | 11.5 |  |  | **+** | 14.4 | 12.0 |  |
| Results are presented as mean $\pm$ SD.  FCW, forward compression wave; BCW, backward compression wave; FEW, forward expansion wave; BEW, backward expansion wave. “Pre” subscription indicates pre-PCI, whereas “post” indicates post-PCI measurements.  WIA peaks have the unit of W.m-2.s-2 . n= 30 for HT and n=32 for no HT (n=1 missing data).  ***Note. * p < .05, ** p < .01, *** p < .001*** | | | | | | | | | | |

**Supplementary Material: Impact of DM**

| **Supplementary Table 2.**  Diabetes Mellitus (DM)$-$no DM Differences of Net Wave Intensity Analysis | | | | | | | | | | |
| --- | --- | --- | --- | --- | --- | --- | --- | --- | --- | --- |
|  | **Variable** | **DM** | **Mean** | **SD** | **P value** | **Variable** | **DM** | **Mean** | **SD** | **p value** |
| **rest** | **FCW_pre_** | **-** | 5.6 | 6.7 | 0.605 | **FCW_post_** | **-** | 7.1 | 5.7 | 0.235 |
|  |  | **+** | 7.0 | 10.1 |  |  | **+** | 5.9 | 5.7 |  |
|  | **BCW_pre_** | **-** | 5.0 | 4.2 | 0.845 | **BCW_post_** | **-** | 5.3 | 4.8 | 0.492 |
|  |  | **+** | 5.7 | 6.8 |  |  | **+** | 6.2 | 4.3 |  |
|  | **FEW_pre_** | **-** | 2.8 | 2.5 | 0.994 | **FEW_post_** | **-** | 4.7 | 4.7 | 0.301 |
|  |  | **+** | 3.9 | 5.3 |  |  | **+** | 3.9 | 4.5 |  |
|  | **BEW_pre_** | **-** | 10.5 | 11.0 | 0.350 | **BEW_post_** | **-** | 10.7 | 10.2 | 0.321 |
|  |  | **+** | 10.4 | 14.4 |  |  | **+** | 16.6 | 18.8 |  |
| **hyperemia** | **FCW_pre_** | **-** | 6.1 | 9.0 | 0.577 | **FCW_post_** | **-** | 10.2 | 6.5 | 0.925 |
|  |  | **+** | 6.7 | 9.5 |  |  | **+** | 12.6 | 14.2 |  |
|  | **BCW_pre_** | **-** | 5.4 | 4.5 | 0.983 | **BCW_post_** | **-** | 11.4 | 10.3 | 0.971 |
|  |  | **+** | 6.1 | 6.5 |  |  | **+** | 11.1 | 8.9 |  |
|  | **FEW_pre_** | **-** | 2.9 | 2.7 | 0.834 | **FEW_post_** | **-** | 6.3 | 5.6 | 0.625 |
|  |  | **+** | 3.5 | 4.8 |  |  | **+** | 5.3 | 6.1 |  |
|  | **BEW_pre_** | **-** | 7.6 | 8.7 | 0.948 | **BEW_post_** | **-** | 13.9 | 10.0 | 0.335 |
|  |  | **+** | 9.5 | 13.8 |  |  | **+** | 16.9 | 13.2 |  |
| Results are presented as mean $\pm$ SD.  FCW, forward compression wave; BCW, backward compression wave; FEW, forward expansion wave; BEW, backward expansion wave. “Pre” subscription indicates pre-PCI, whereas “post” indicates post-PCI measurements.  WIA peaks have the unit of W.m^-2^.s^-2^ . n= 21 for DM and n=42 for no DM. | | | | | | | | | | |

**Supplementary Material: Impact of Sex**

| **Supplementary Table 3.** Female$-$Male Sex Differences of Net Wave Intensity Analysis | | | | | | | | | | |
| --- | --- | --- | --- | --- | --- | --- | --- | --- | --- | --- |
|  | **Variable** | **Sex** | **Mean** | **SD** | **p value** | **Variable** | **Sex** | **Mean** | **SD** | **p value** |
| **rest** | **FCW_pre_** | **F** | 3.2 | 2.5 | 0.208 | **FCW_post_** | **F** | 7.2 | 5.4 | 0.500 |
|  |  | **M** | 6.8 | 8.6 |  |  | **M** | 6.6 | 5.8 |  |
|  | **BCW_pre_** | **F** | 4.5 | 4.9 | 0.643 | **BCW_post_** | **F** | 4.4 | 5.4 | 0.053 |
|  |  | **M** | 5.4 | 5.3 |  |  | **M** | 5.9 | 4.5 |  |
|  | **FEW_pre_** | **F** | 1.7 | 1.1 | 0.121 | **FEW_post_** | **F** | 5.7 | 4.9 | 0.186 |
|  |  | **M** | 3.5 | 4.0 |  |  | **M** | 4.1 | 4.5 |  |
|  | **BEW_pre_** | **F** | 6.1 | 4.1 | 0.224 | **BEW_post_** | **F** | 8.7 | 7.7 | 0.259 |
|  |  | **M** | 11.5 | 13.1 |  |  | **M** | 13.6 | 14.8 |  |
| **hyperemia** | **FCW_pre_** | **F** | 5.1 | 6.3 | 0.630 | **FCW_post_** | **F** | 9.4 | 6.7 | 0.606 |
|  |  | **M** | 6.6 | 9.6 |  |  | **M** | 11.4 | 10.3 |  |
|  | **BCW_pre_** | **F** | 6.7 | 5.4 | 0.266 | **BCW_post_** | **F** | 8.8 | 6.7 | 0.630 |
|  |  | **M** | 5.4 | 5.2 |  |  | **M** | 11.9 | 10.3 |  |
|  | **FEW_pre_** | **F** | 3.0 | 3.6 | 0.668 | **FEW_post_** | **F** | 7.4 | 8.2 | 0.457 |
|  |  | **M** | 3.1 | 3.5 |  |  | **M** | 5.6 | 5.1 |  |
|  | **BEW_pre_** | **F** | 7.6 | 6.1 | 0.489 | **BEW_post_** | **F** | 10.9 | 4.9 | 0.274 |
|  |  | **M** | 8.3 | 11.4 |  |  | **M** | 15.9 | 12.0 |  |
| Results are presented as mean $\pm$ SD.  FCW, forward compression wave; BCW, backward compression wave; FEW, forward expansion wave; BEW, backward expansion wave. “Pre” subscription indicates pre-PCI, whereas “post” indicates post-PCI measurements.  WIA peaks have the unit of W.m^-2^.s^-2^ . n= 12 for female and n=51 for male, note the main study group have 2 patients in common (both are male), each of whom had 2 revascularized vessels. | | | | | | | | | | |

| Supplementary Table 4. Correlations between Amplitudes of Accelerative WI Peaks and Other Hemodynamic Indices | | | | | | | |
| --- | --- | --- | --- | --- | --- | --- | --- |
|  | FFR_pre_ | CFVR_pre_ | hSR_pre_ | bMR_pre_ | hMR_pre_ | bAPV_pre_ | hAPV_pre_ |
| FCW_pre_ rest | 0.220 | 0.025 | -0.308* | -0.445*** | -0.319* | 0.490*** | 0.382** |
| BEW_pre_ rest | 0.310* | 0.178 | -0.418*** | -0.285* | -0.322* | 0.475*** | 0.435*** |
| FCW_pre_ hype | 0.338** | 0.121 | -0.441*** | -0.350** | -0.309* | 0.560*** | 0.534*** |
| BEW_pre_ hype | 0.484*** | 0.311* | -0.550*** | -0.161 | -0.250* | 0.431*** | 0.545*** |
|  | FFR_post_ | CFVR_post_ | hSR_post_ | bMR_post_ | hMR_post_ | bAPV_post_ | hAPV_post_ |
| FCW_post_ rest | 0.203 | -0.124 | -0.321* | -0.426*** | -0.327** | 0.572*** | 0.463*** |
| BEW_post_ rest | -0.048 | -0.343** | -0.106 | -0.493*** | -0.290* | 0.519*** | 0.236 |
| FCW_post_ hype | 0.295* | 0.115 | -0.465*** | -0.337** | -0.345** | 0.468*** | 0.499*** |
| BEW_post_ hype | 0.063 | 0.058 | -0.279* | -0.309* | -0.363** | 0.398** | 0.444*** |
| FCW, forward compression wave; BCW, backward compression wave; FEW, forward expansion wave; BEW, backward expansion wave; FFR, fractional flow reserve; CFVR, coronary flow velocity reserve; hSR, hyperemic stenosis resistance; bMR, basal microvascular resistance; hMR, hyperemic microvascular resistance; bAPV, basal average peak velocity; hAPV, hyperemic average peak velocity “Pre” subscription indicates pre-PCI, whereas “post” indicates post-PCI measurements.  *Note. * p < .05, ** p < .01, *** p < .001* | | | | | | | |

**Supplementary Material: Impact of Heart Rate
Impact of Heart Rate on WIA
and Present Observations**The heart rate-adjusted and unadjusted WIA peak amplitudes were almost perfectly correlated (e.g., hyperemic FCWpost adjusted – unadjusted: r = 0.984, p < 0.001; BEWpost adjusted – unadjusted: r = 0.975, p < 0.001), indicating minimal effect of heart rate on the results. Consistently, post-PCI heart rate itself showed no significant correlation with WI amplitudes, whereas pre-PCI heart rate had some moderate correlations; Supplementary Table 5). Importantly, the heart rate-adjusted WI profiles compared between the discordant and concordant groups yielded parallel results with the main analysis (Supplementary Table 6). Thus, the group differences in FCW and BEW were not driven by heart rate differences.

| Supplementary Table 5. Correlations between Heart Rate (HR) and Amplitudes of WIA Peaks | | |
| --- | --- | --- |
|  | **HR_pre_ rest** | **HR_pre_ hype** |
| FCW_pre_ rest | 0.275* | 0.299* |
| BEW_pre_ rest | 0.152 | 0.169 |
| FCW_pre_ hype | 0.383** | 0.402** |
| BEW_pre_ hype | 0.078 | 0.320* |
|  | **HR_post_ rest** | **HR_post_ hype** |
| FCW_post_ rest | 0.054 | 0.064 |
| BEW_post_ rest | 0.092 | 0.122 |
| FCW_post_ hype | -0.044 | -0.021 |
| BEW_post_ hype | 0.167 | 0.115 |
| FCW, forward compression wave; BCW, backward compression wave; FEW, forward expansion wave; BEW, backward expansion wave. “Pre” subscription indicates pre-PCI, whereas “post” indicates post-PCI measurements.  *Note. * p < .05, ** p < .01, *** p < .001* | | |

| Supplementary Table 6. Heart Rate-adjusted Peaks of FCW and BEW of Residual Low FFR (post PCI) | | | | | |
| --- | --- | --- | --- | --- | --- |
|  | FFR* | N | Mean/n | SD/% | p value |
| aFCW_pre_ rest | **> 0.8** | 53 | 891.577 | 1148.296 | 0.771 |
|  | **≤ 0.8** | 10 | 540.939 | 323.590 |  |
| aBEW_pre_ rest | **> 0.8** | 53 | 1515.840 | 1824.929 | 0.903 |
|  | **≤ 0.8** | 10 | 1179.909 | 720.943 |  |
| aFCW_pre_ hype | **> 0.8** | 53 | 980.676 | 1396.846 | 0.173 |
|  | **≤ 0.8** | 10 | 362.123 | 168.109 |  |
| aBEW_pre_ hype | **> 0.8** | 53 | 1263.343 | 1699.684 | 0.211 |
|  | **≤ 0.8** | 10 | 678.446 | 458.050 |  |
| aFCW_post_ rest | **> 0.8** | 53 | 1011.795 | 884.179 | 0.858 |
|  | **≤ 0.8** | 10 | 809.546 | 506.220 |  |
| aBEW_post_ rest | **> 0.8** | 53 | 1967.372 | 2427.125 | 0.933 |
|  | **≤ 0.8** | 10 | 1480.748 | 1084.655 |  |
| aFCW_post_ hype | **> 0.8** | 53 | 1769.180 | 1648.501 | ***0.011**** |
|  | **≤ 0.8** | 10 | 848.866 | 683.307 |  |
| aBEW_post_ hype | **> 0.8** | 53 | 2332.238 | 1792.358 | ***0.033**** |
|  | **≤ 0.8** | 10 | 1394.328 | 1169.634 |  |
| aFCW, heart-rate adjusted forward compression wave; aBCW, heart-rate adjusted backward compression wave; aFEW, heart-rate adjusted forward expansion wave; aBEW, heart-rate adjusted backward expansion wave.  **post-PCI FFR*  *Note. * p < .05, ** p < .01, *** p < .001* | | | | | |
